# Sticky swinging arm dynamics: studies of an acyl carrier protein domain from the mycolactone polyketide synthase

**DOI:** 10.1042/BCJ20160041

**Published:** 2016-04-08

**Authors:** Steven Vance, Olga Tkachenko, Ben Thomas, Mona Bassuni, Hui Hong, Daniel Nietlispach, William Broadhurst

**Affiliations:** * Department of Biochemistry, University of Cambridge, 80 Tennis Court Road, Cambridge CB2 1GA, U.K.; † Crescendo Biologics Ltd, Meditrina Building 260, Babraham Research Campus, Cambridge CB22 3AT, U.K.; ‡ Department of Chemistry, University of Oxford, South Parks Road, Oxford OX1 5QY, U.K.

**Keywords:** acyl carrier protein, mycolactone, NMR spectroscopy, 4′-phosphopantetheine, type I polyketide synthase

## Abstract

Type I modular polyketide synthases (PKSs) produce polyketide natural products by passing a growing acyl substrate chain between a series of enzyme domains housed within a gigantic multifunctional polypeptide assembly. Throughout each round of chain extension and modification reactions, the substrate stays covalently linked to an acyl carrier protein (ACP) domain. In the present study we report on the solution structure and dynamics of an ACP domain excised from MLSA2, module 9 of the PKS system that constructs the macrolactone ring of the toxin mycolactone, cause of the tropical disease Buruli ulcer. After modification of *apo* ACP with 4′-phosphopantetheine (Ppant) to create the *holo* form, ^15^N nuclear spin relaxation and paramagnetic relaxation enhancement (PRE) experiments suggest that the prosthetic group swings freely. The minimal chemical shift perturbations displayed by Ppant-attached C_3_ and C_4_ acyl chains imply that these substrate-mimics remain exposed to solvent at the end of a flexible Ppant arm. By contrast, hexanoyl and octanoyl chains yield much larger chemical shift perturbations, indicating that they interact with the surface of the domain. The solution structure of octanoyl-ACP shows the Ppant arm bending to allow the acyl chain to nestle into a nonpolar pocket, whereas the prosthetic group itself remains largely solvent exposed. Although the highly reduced octanoyl group is not a natural substrate for the ACP from MLSA2, similar presentation modes would permit partner enzyme domains to recognize an acyl group while it is bound to the surface of its carrier protein, allowing simultaneous interactions with both the substrate and the ACP.

## Abstract (precis)

When covalently linked to an acyl carrier protein (ACP) and loaded with acyl substrate-mimics, some 4′-phosphopantetheine prosthetic group arms swing freely, whereas others stick to the protein surface, suggesting a possible mode of interaction with enzyme domains during polyketide biosynthesis.

## INTRODUCTION

Type I modular polyketide synthases (PKSs) are large, multi-domain complexes responsible for generating natural products with a spectrum of medically important activities, including antibiotic, anticancer, antifungal, antitumour and immunosuppressive properties [1]. Like type I fatty acid synthases (FASs), these systems consist of a series of covalently linked enzymes that extend a polyketide substrate by two carbon atoms and modify the functionality of the newly added building block via reactions at the β-ketone site. In FAS systems the substrate is cycled repeatedly between a single set of enzymes to produce long, saturated acyl chains, but in the majority of modular PKS systems each extension step is carried out in sequence by a distinct set or ‘module’ of enzymes.

**Figure 1:**
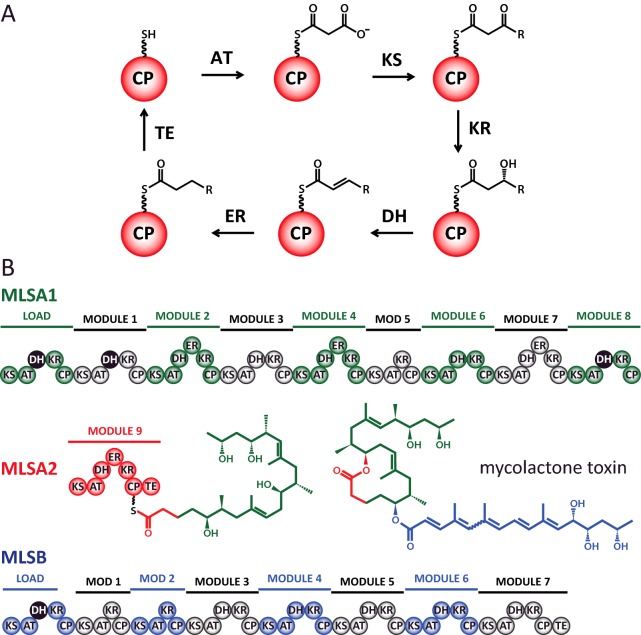
Reaction scheme and module organization for the mycolactone PKS system (**A**) Catalytic cycle for domains from the MLSA2 module. (**B**) Module organization for the three subunits of the mycolactone PKS system (MLSA1, MLSA2 and MLSB). The product of MLSA2 is shown attached to its carrier protein domain. The structure of mycolactone is colour coded to match the subunits responsible for synthesizing each segment. DH domains predicted to be inactive are shaded black.

PKS extension modules comprise three domains essential for constructing the product chain [2]: a small (∼10 kDa) acyl carrier protein (ACP) to which the polyketide substrate is tethered via thioester linkage to a 4′-phosphopantetheine (Ppant) prosthetic group; an acyltransferase (AT), which selects an appropriate extender unit (commonly malonate or methylmalonate as their coenzyme A thioesters) for loading on to the ACP; and a ketosynthase (KS), which accepts a polyketide chain from a previous module and attaches the new extender unit by catalysing a decarboxylative condensation reaction (Figure 1A). To introduce chemical diversity into the polyketide product, modules can contain additional enzyme domains [2]: a ketoreductase (KR) that reduces the β-ketone group to an alcohol and may also epimerize the adjacent α-centre; a dehydratase (DH), which eliminates the β-hydroxy to form an α-β double bond; and an enoyl reductase (ER) that reduces the resulting alkene, producing a saturated β-methylene group (Figure 1A). The terminal module of a PKS system normally contains a thioesterase (TE) domain, which promotes release and often cyclization of the substrate. The length and functionality of the final product is therefore defined by the number, order and domain composition of modules within the system [1].

The intuitive, linear, assembly-line nature of modular PKS and similarly configured non-ribosomal peptide synthetase (NRPS) systems make them attractive targets for combinatorial biosynthesis and synthetic biology strategies [3,4]. Although numerous new compounds have been generated by such approaches, engineered PKS multi-enzyme complexes often display reduced activity or result in undesirable product mixtures [5,6]. A major limitation in overcoming such deficiencies is a poor understanding of the interactions that occur within and between modules. The role of the ACP is central to this question, since it must present covalently tethered substrates to the active sites of each enzymatic domain within its module, as well as a KS or a TE in the subsequent module [7,8]. However, whether the ACP plays an active role in this process or merely restricts free diffusion of the substrate has not yet been fully established for type I PKS systems [9,10].

Transitions between different module configurations may restrict the access of an ACP to a subset of partner domains during different phases of the reaction cycle [10], but from the point of view of the carrier protein three potential mechanisms for identifying the appropriate active site can be envisaged: (i) substrate-mediated modifications of ACP structure to promote enzyme-specific protein–protein interactions; (ii) recognition of a combination of the ACP surface and substrate chemistry; or (iii) pure recognition of substrate chemistry by the catalytic domain in the absence of specific interactions with the ACP. Identifying which of these mechanisms is used to regulate interactions between substrate-loaded ACPs and the various catalytic domains will have important consequences for rational engineering of PKS assembly lines to produce novel polyketide scaffolds.

Type II PKS systems consist of discrete domains, so their acylated ACP domains can only interact with partner enzymes in *trans*. Carrier proteins of this sort typically protect complex substrate chains from contact with the solvent by sequestering them inside a hydrophobic pouch to minimize premature hydrolysis and unwanted side reactions [9,11]. In the case of the *Streptomyces coelicolor* FAS ACP, solution structures for a complete set of C_6_ reaction intermediates demonstrate that although the global fold of the domain is retained, the conformations of the thioester, the Ppant moiety and nearby regions of the protein are subtly different [12]. These structural changes could assist in selecting the correct active site for the next step in the catalytic cycle. However, the type I FAS and PKS ACP domains studied to date do not appear to bury their substrates [13–16], so the same mode of conformational programming may not apply. Similarly, the ‘switchblade’ [17] or ‘chain-flipping’ [11] paradigms required to explain how a partner enzyme can gain access to a reduced acyl chain that is sequestered inside the hydrophobic core of a type II ACP domain may not be relevant to type I systems if ACP-tethered substrates are continuously on display, sheltered by the quaternary architecture of the surrounding module. Current investigations of substrate-loaded NRPS aryl and peptidyl carrier protein domains from the Type I yersiniabactin [18] and the Type II pyoluteorin [19] systems are consistent with a more delicate mechanism: the distribution of charge across the surface of the domain is modulated by interactions with both the Ppant arm and the substrate in ways that could favour productive encounters with specific partners.

Recent cryo-electron microscopy (cryo-EM) studies on the fifth chain-extension module from the pikromycin PKS (Pik module 5) suggest that positioning of the ACP within a type I module may be driven by the identity of the substrate attached to the Ppant arm [20,21]. ACP domains modified with substrate mimics were found to dock on to the appropriate enzymatic domain for the ensuing reaction within the module's catalytic cycle, although predominantly at distances too remote for the substrate to access the relevant active sites [22]. Further, loading a polyketide chain that mimicked the module's final product caused the ACP to protrude from the base of the module at a position thought to be suitable for downstream transfer of the substrate to a subsequent module or TE. Of the three mechanisms for ACP partner selection suggested above, these results favour the second, as in most cases both protein–protein interfaces and correct substrate chemistry appear to be necessary for progression through the catalytic cycle [10,20,21].

The PKS responsible for production of the *Mycobacterium ulcerans* toxin mycolactone, the molecular cause of Buruli ulcer [23], presents a particularly attractive system in which to study the intricacies of modular PKS systems. Despite significant variations in the length and chemistry of the substrate encountered at each step, the mycolactone synthases display a remarkably high sequence identity (>95%) between equivalent domains across 16 chain extension modules found in three protein chains: MLSA1, MLSA2 and MLSB (Figure 1B) [24,25]. Such a high degree of similarity between modules suggests either that enzymatic domains from the mycolactone PKS are less discriminating (and may therefore be good candidates for combinatorial biosynthesis and synthetic biology), or that the few residues which do vary are specific for particular features of natural substrates.

As a first step towards the biophysical characterization of interactions between carrier protein and partner enzyme domains from a canonical type I PKS module that contains a complete reductive loop and a chain-releasing TE domain, we report here studies of the solution structure and dynamics of mACP_9_, the ACP domain from MLSA2, module 9 of the PKS system that constructs the macrolactone ring of mycolactone (Figure 1B) [25]. We demonstrate that different substrate-mimics interact with the surface of the ACP in distinct ways that could play a role in the recognition of partner domains.

## MATERIALS AND METHODS

### Expression and purification of *apo* mACP_9_

The mACP_9_ sequence from the *mlsA2* gene from *Mycobacterium ulcerans* (Uniprot: Q6MZA5; residues 2050–2140) cloned into pET28 (EMD Millipore) was transformed into competent *Escherichia coli* Tuner (DE3) cells (EMD Millipore). His_6_-tagged mACP_9_ was expressed at 15°C for 16 h in 1 litre of LB or M9 medium, prepared according to standard protocols [26], with 30 μg/ml kanamycin (Sigma) for selection and 1 mM isopropyl β-D-1-thiogalactopyranoside (IPTG) (Sigma) for induction. Isotopically labelled samples were prepared by supplementing M9 with ^15^N ammonium chloride (Sigma) and ^13^C_6_-D-glucose (Cambridge Isotope Laboratories). Cells were harvested at a 600 nm absorbance of 0.6, resuspended in lysis buffer (50 mM sodium hydrogen phosphate, 300 mM sodium chloride, pH 8.0) with 5 mM imidazole, 2.5 units/ml benzonase nuclease (EMD Millipore) and Sigmafast EDTA-free protease inhibitor cocktail (Sigma) and lysed using an Emulsiflex C5 homogeniser (Glen Creston).

The clarified lysate was passed through Ni-NTA resin (Qiagen), washed twice with lysis buffer containing 30 mM imidazole and eluted with lysis buffer containing 300 mM imidazole. The eluted protein was exchanged into phosphate buffer (50 mM sodium hydrogen phosphate, 150 mM sodium chloride, pH 7.5) and the His_6_-tag was cleaved using restriction grade thrombin (EMD Millipore). The released mACP_9_ was further purified by size exclusion chromatography using an Äkta Purifier 10 system and a Superdex 75 10/300 column (GE Healthcare) in phosphate buffer. The sample was concentrated using 5000 MWCO Vivaspin 20 columns (Sartorius Stedim). The expression and purification was monitored by SDS/PAGE (NuPAGE) 4–12% Bis-Tris gels (Life Technologies) stained with InstantBlue (Expedeon). The identity of the sample was confirmed by electrospray injection mass spectrometry (ESI MS; Supplementary Table S1 and Supplementary Figure S1).

### Preparation of covalently modified forms of mACP_9_

#### Co-expression with Sfp to make ^15^N-labelled holo mACP_9_

The mACP_9_/pET28 plasmid and a pSU2718 plasmid [27] with the *Sfp* gene cloned between the *Nde*I and *Sal*I sites were transformed into competent *E. coli* Tuner (DE3) cells (EMD Millipore). Apart from addition of 34 μg/ml chloramphenicol (Sigma) to growth media for selection of the pSU2718 plasmid, expression and purification of the *holo* protein was identical with that of the *apo* protein described above. The extent of modification of mACP_9_ was monitored by ESI MS (Supplementary Figure S2), and was complete in all cases taken forward for further study.

#### Sfp-based addition of 4′-phosphopantetheine and acyl-phosphopantetheine groups

*In vitro* loading reactions were performed on 500 μM *apo* ACP_9_ samples by incubation with 5 μM recombinant Sfp [28], 2 mM coenzyme A or its derivatives malonyl, butyryl, 2-butenoyl, β-hydroxybutyryl, acetoacetyl, hexanoyl or octanoyl CoA (all Sigma) in pH 7.5 phosphate buffer with 10 mM magnesium chloride at 20°C for 1 h. 10 mM DTT was added when handling coenzyme A and *holo* mACP_9_ to prevent disulfide bond formation between exposed thiol groups. Samples were subjected to size exclusion chromatography as described above prior to further analysis. In each case, the identity and extent of modification was monitored by ESI MS (Supplementary Figures S3–S9).

#### Methanthiosulfonate-based modification of the 4′-phosphopantetheine thiol

(1-Oxyl-2,2,5,5-tetramethyl-∆3-pyrroline-3-methyl) methane-thiosulfonate (MTSL) or (1-acetoxy-2,2,5,5-tetramethyl-∆3-pyrroline-3-methyl) methanethiosulfonate (ATSL) (Toronto Research Chemicals), both dissolved in DMSO, were added to *holo* mACP9 in a 10-fold molar excess at less than 1% of the sample volume, then incubated at 20°C for 16 h. *n*-Propyl methanethiosufonate (PMTS) oil (Toronto Research Chemicals) was added directly to *holo* mACP9 at a 20-fold molar excess and at less than 0.2% of the final sample volume. All samples were incubated at 20°C for 16 h and then subjected to size exclusion chromatography as described above prior to further analysis. Full labelling was confirmed by ESI MS (Supplementary Figures S10–S12).

### NMR experiments for assignment and distance restraints

All samples for NMR spectroscopy were prepared at concentrations of 200–800 μM in phosphate buffer supplemented with 10% D_2_O (Sigma) and 0.0025% 3,3,3-trimethylsilylpropionate (Sigma) in 5 mm Ultra-Imperial grade NMR tubes (Wilmad) to a final volume of 600 μL. 10 mM DTT was added to *holo* samples. [^1^H,^15^N]-HSQC, ^15^N-TOCSY-HSQC, ^15^N-NOESY-HSQC, HNCA, HNCOCA, HNCACB and CBCA(CO)NH spectra were recorded at 283 K on a Bruker DRX500 spectrometer equipped with a *z*-shielded gradient triple resonance probe, using standard procedures [29]. ^13^C-NOESY-HSQC spectra were recorded at 283 K on a Bruker Avance DRX800 spectrometer equipped with a 5 mm TXI CryoProbe. All NMR spectra were processed using the Azara package (www.ccpn.ac.uk/azara), then analysed and assigned using CcpNmr Analysis software [30]. To compare resonance positions in [^1^H,^15^N]-HSQC spectra of different mACP_9_ species, average chemical shift differences were determined using the formula Δ*δ*_av_={0.5(Δ*δ*_H_)^2^ + 0.1(Δ*δ*_N_)^2^}^0.5^ [31]. The threshold for a significant shift change (0.042 ppm) was calculated as twice the S.D. of the differences in all data sets remaining after eliminating outliers with differences greater than two S.D. from the initial mean.

### Determination of solution structures for *apo* and octanoyl-mACP_9_

All structures of *apo* mACP_9_ were calculated from extended templates by simulated annealing using ARIA 2.3 [32], with manual screening of ambiguous restraints. Backbone *φ* and *ψ* dihedral angle restraints were determined from chemical shifts using the DANGLE program [33]. NOE distance restraints generated by the resonance assignment process and dihedral angle restraints were fed as input. Nine iterations were performed, each determining 20 structures, except for the final round, in which 100 were calculated, followed by refinement in explicit solvent for the 20 lowest energy structures, all of which were selected for the final ensemble, which contains no distance violations >0.5 Å (1 Å=0.1 nm) and includes >97% of residues in the ‘most favoured’ and ‘allowed’ regions of the Ramachandran plot*.* The atomic coordinates of the final ensemble for *apo* mACP_9_ were deposited in the Protein Data Bank under ID code 5HVC; the corresponding NMR assignments were deposited in the Biological Magnetic Resonance Data Bank under accession code 30007.

Structures of octanoyl-mACP_9_ were calculated following the method described above for the *apo* form. Ser^2096^ was replaced with a modified serine residue in which an octanoyl-phosphopantetheine group replaced the H^
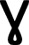
^ atom. Topology files for the modified serine residue were created using the programs ACPYPE and ANTECHAMBER [34–36]. The atomic coordinates of the final ensemble for octanoyl-mACP_9_ were deposited in the Protein Data Bank under ID code 5HV8; the corresponding NMR assignments were deposited in the Biological Magnetic Resonance Data Bank under accession code 30006.

### ^15^N nuclear spin relaxation experiments

^15^N nuclear spin relaxation experiments were recorded using standard procedures [29] at 283K on a Bruker DRX500 spectrometer. ^15^N *T*_1_ delays (ms): 10, 50, 100, 150, 250, 400, 550, 700, 850, 1000. ^15^N *T*_2_ delays (ms): 14.4, 28.8, 43.2, 57.6, 72.0, 86.4, 100.8, 155.2. The heteronuclear NOE reference and saturation experiments were carried out in duplicate to allow an estimation of the error. An initial *τ*_c_ estimate was obtained from the *R*_2_/*R*_1_ ratios for each residue [37]; the same procedure was used to make site specific estimates of the local rotational correlation time *τ*_eff_. The relaxation parameters were analysed with version 4 of the Modelfree program [38] using the strategy described by Mandel et al. [39]. H^N^─N bond vectors from the solution structure of *apo* mACP_9_ were used for anisotropic diffusion tensor modelling of relaxation data for both the *apo* and *holo* forms.

### Paramagnetic relaxation enhancement experiments

^1^H^N^ *T*_2_ experiments [40] were recorded with delays (ms): 0.002, 8, 16 (reference); 0.002, 8, 16, 24 (MTSL-labelled). The reference sample was prepared by reducing the paramagnetic MTSL with a 5-fold molar excess of ascorbic acid (Sigma) from a 200 mM stock solution, added directly to the MTSL-labelled mACP_9_ NMR sample and incubated at 25°C for 2 h.

### Circular dichroism spectroscopy

Thermal denaturation profiles were obtained by monitoring the molar ellipticity [*θ*] at 220 nm on an Aviv Model 410 circular dichroism spectrometer. Samples of mACP_9_ species were prepared at 0.1 mg/ml in 20 mM sodium hydrogen phosphate, 50 mM sodium fluoride, pH 7.5. [*θ*] was recorded at 1°C increments ranging from 20°C to 95°C. The unfolded state percentage was calculated using the formula *F*(*T*)={([*θ*]_max_ − [*θ*]*_T_*)/([*θ*]_max_ − [*θ*]_min_)} × 100, where [*θ*]_max_ is the maximum observed value of [*θ*], [*θ*]_min_ is the minimum observed value and [*θ*]*_T_* is the [*θ*] recorded at temperature, *T*. The melting temperature *T*_m_ was estimated from the inflexion point of the normalized melting curves. Reported *T*_m_ values are the mean for three repeat experiments.

## RESULTS

### Expression and purification of mACP_9_ species

mACP_9_, a protein fragment spanning residues 2050–2140 of the MLSA2 subunit, was expressed with an N-terminal His_6_-tag, as described in Materials and Methods. Following purification using nickel affinity chromatography, the fusion tag was removed by thrombin cleavage, leaving four non-native amino acid residues originating from the expression vector (GSHM-) at the N-terminus of the construct. Analytical size exclusion chromatography indicated that *apo* mACP_9_ is monomeric in solution (Supplementary Figure S13). Phosphopantetheinylation of mACP_9_ was carried out *in vivo* by co-expression with Sfp, a broad specificity phosphopantetheinyl transferase [28], resulting in full conversion from the *apo* to the *holo* form according to ESI MS (Supplementary Figure S2).

### Solution structure of *apo* mACP_9_

**Figure 2:**
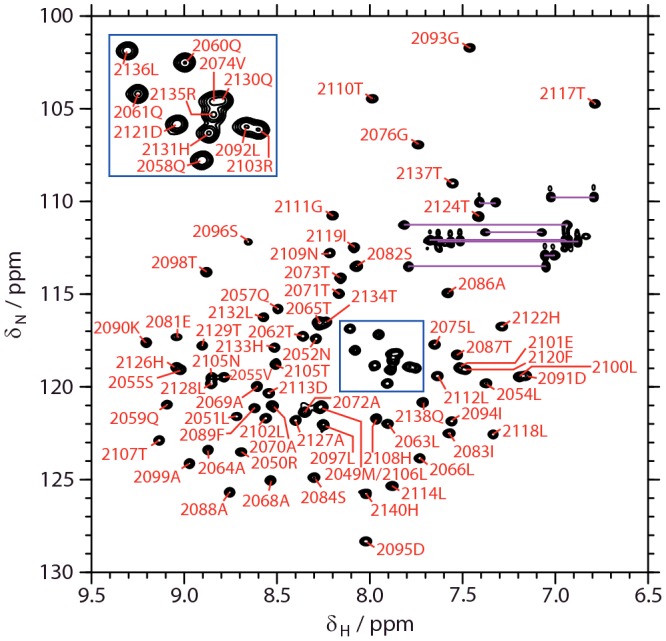
NMR spectroscopy [^1^H,^15^N]-HSQC spectrum of *apo* mACP_9_, showing residue assignments for backbone amide sites. Pairs of resonances from side-chain amide sites are connected using magenta lines. Assignments for closely spaced signals are displayed in the inset panel.

The [^1^H,^15^N]-HSQC spectrum of *apo* mACP_9_ displayed well-resolved, dispersed signals indicative of a structured protein (Figure 2). Eighty-three of the 84 expected backbone amide resonances were assigned; no signal was detected for His^2077^, which is located in a flexible surface exposed loop. Single resonances were observed for each backbone and side-chain amide site, suggesting that the domain adopts a unique conformation in solution.

**Figure 3:**
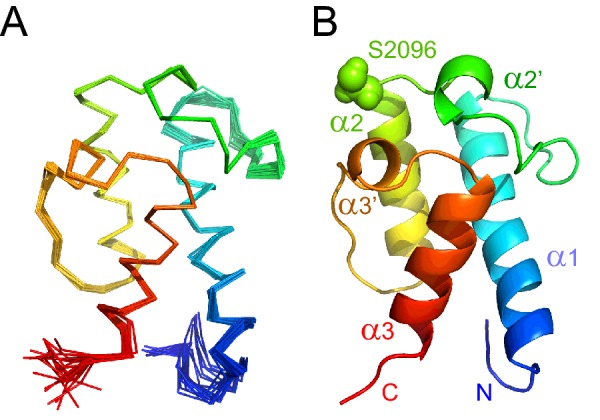
Solution structure of *apo* mACP_9_ (**A**) Backbone overlay for the final ensemble of 20 lowest energy structures, coloured from blue at the N-terminus to red at the C-terminus (5HVC). (**B**) Ribbon representation of the lowest energy structure.

A total of 2953 NOE-derived distance restraints, of which 1424 were unambiguously assigned, were used to calculate the solution structure of *apo* mACP_9_. After water refinement, the final ensemble comprising the 20 lowest energy structures (Figure 3A) has a backbone coordinate RMSD of 0.6 Å for residues 2050–2140, reducing to 0.4 Å over the sections of regular secondary structure; further statistics are summarized in Table 1. The compact, right-hand twisted helical bundle (Figure 3B) is typical of carrier proteins from PKS, FAS and NRPS systems [9]. The three main α helices (α1: 2056–2075; α2: 2096–2110 and α3: 2125–2137) are interspersed with two short helical turns (α2′: 2089–2092 and α3′: 2118–2121). The main helices span the long axis of the domain, with α1 antiparallel to α2 and α3, whereas the shorter turns are approximately perpendicular to the long axis. Ser^2096^, the attachment point for Ppant modification, is positioned at the opposite end of the domain to the two termini. The side chain of Ser^2096^ projects out towards the solvent at the beginning of helix α2.

**Table 1:** Restraints and statistics for the solution structures of *apo* and octanoyl-mACP_9_

	*apo* mACP_9_	Octanoyl-mACP_9_
NOE-based distance restraints		
Intra-residue, sequential	705	406
Medium range (2 ≤ | *i* − *j* | ≤ 5)	244	280
Long range (| *i* − *j* | > 5)	183	330
Ambiguous	1707	454
Total	2839	1949
Other restraints		
Hydrogen bond restraints	0	0
*φ* + *ψ* dihedral angles restraints	167	167
Coordinate precision*		
Backbone RMSD (Å)	0.61±0.11	0.96±0.19
Heavy atom RMSD (Å)	1.17±0.18	1.46±0.24
Consistency (structure compared with restraints)		
RMSD (Å) from experimental distance restraints	0.032±0.002	0.036±0.006
RMSD (°) from experimental dihedral angle restraints	1.97±0.08	1.12±0.08
Ramachandran plot		
Most favoured	89.3%	91.1%
Allowed regions	8.1%	8.0%
Generously allowed regions	1.2%	0.7%
Disallowed regions	1.4%	0.3%
WHATIF structure *Z*-scores		
First generation packing quality	1.09±0.63	1.03±0.59
Second generation packing quality	6.36±1.72	5.63±1.61
Ramachandran plot appearance	−3.60±0.25	−3.31±0.32
*χ*_1_/*χ*_2_ rotamer normality	−4.04±0.35	−5.38±0.51
Backbone conformation	−0.15±0.42	−0.10±0.02

*Coordinate precision, Ramachandran statistics and *Z*-scores were determined between residues 2050 and 2140.

The core of the calculated structure contains closely packed hydrophobic side chains, with no evidence for the interior cavities observed in type II ACP structures [9]. The exterior surface is predominately composed of hydrophilic side chains, the only exception being a small nonpolar patch adjacent to the attachment serine, produced by Phe^2120^ from turn α3′ and two solvent-exposed leucine side chains from helix α2 (Leu^2097^ and Leu^2100^). The nonpolar surface of the α2 ‘recognition helix’ is a common distinction observed between type I and type II ACPs, for which the equivalent region, proposed to be critical for interactions with partner enzymes, is rich in acidic residues [9]. In the mACP_9_ structure, helix α2 contains only one acidic residue, Glu^2101^; the majority of charged residues, which are predominantly basic, are located on a face created by α2, α3′ and α3, whereas the opposing α1 face is relatively uncharged (Supplementary Figure S14).

### *Holo* and acyl-loaded forms of mACP_9_

**Figure 4:**
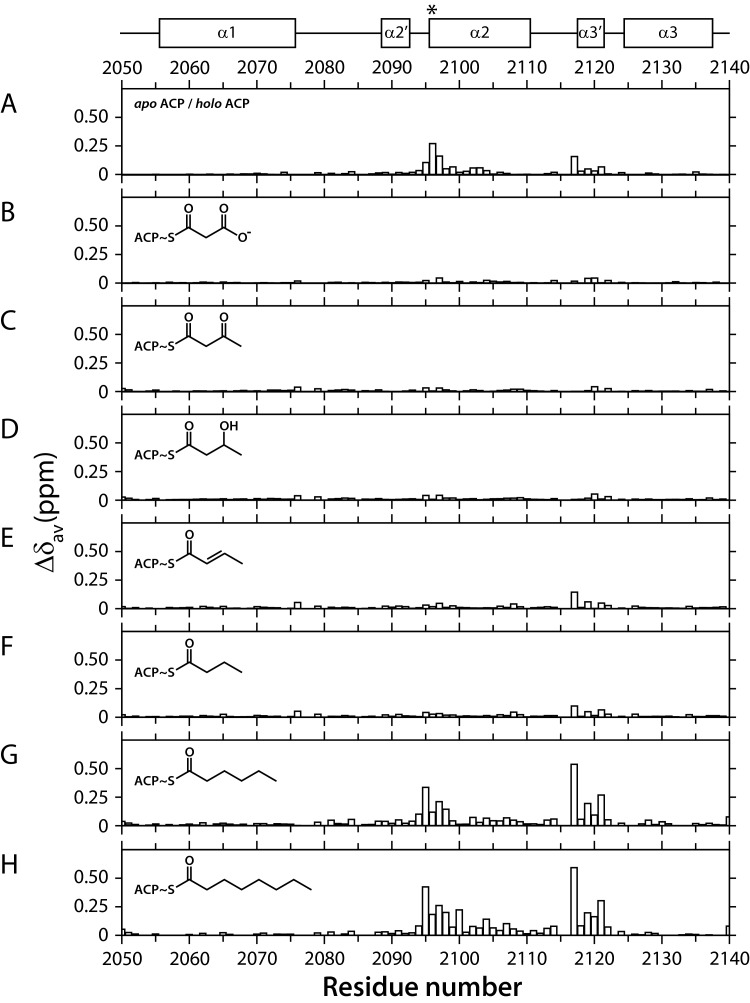
Chemical shift differences between modified forms of mACP_9_ Underneath a schematic defining the boundaries of α-helices in the structure of *apo* mACP_9_, average chemical shift differences, Δ*δ*_av_, are plotted as a function of residue number. Differences are shown between *holo* mACP_9_ and the (**A**) *apo*; (**B**) malonyl; (**C**) acetoacetyl; (**D**) hydroxybutyryl; (**E**) buten-2-oyl; (**F**) butyryl; (**G**) hexanoyl; and (**H**) octanoyl forms. Ser^2096^, the point of attachment for the prosthetic group, is indicated with an asterisk.

Backbone assignments for the *apo* form were transferred to experiments recorded on a *holo* mACP_9_ sample and verified using NOE connections. As noted for the *apo* form above, the number of signals detected was consistent with population of a single conformational state. Two additional peaks in the [^1^H,^15^N]-HSQC spectrum of *holo* mACP_9_ were identified as amide signals from N^4^ and N^8^ in the Ppant arm (Supplementary Figure S15). The profile of average ^1^H^N^/^15^N chemical shift differences shown in Figure 4A demonstrates that the majority of backbone amide sites experience only minor perturbations in their electronic environment (<0.03 ppm), suggesting that the structure of mACP_9_ changes little between the *apo* and *holo* forms. Significant differences are observed for residues adjacent to the attachment serine (Ile^2094^-Asn^2104^) and for sites towards the N-terminus of the nearby α3′ turn (Thr^2117^-Asp^2121^).

In type II ACP domains, the α3′ turn can undergo a minor structural rearrangement upon conversion to the *holo* form [9], but analysis of ^15^N- and ^13^C-separated NOESY spectra recorded on both *apo* and *holo* states of mACP_9_ revealed no significant differences, even for residues that display Δ*δ*_av_ values >0.05 ppm, indicating that the backbone conformations of the two species remain very similar. In a comparison of *apo* and *holo* forms of the type I ACP from module 6 of the DEBS system, similar patterns of chemical shift perturbation were proposed to be a consequence of proximity to the attachment site, resulting from transient interactions with the dynamic Ppant moiety [13]. Interestingly, for mACP_9_ the magnitude of shift change does not have a simple relationship to distance from the attachment serine. For example, in the *apo* mACP_9_ structure the backbone amide proton (H^N^) of Thr^2117^ is 10.5 Å from the side-chain O^
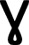
^ atom of Ser^2096^ and possesses a Δ*δ*_av_ of 0.16 ppm, whereas Lys^2090^ H^N^, which is 10.1 Å away, shows a Δ*δ*_av_ of only 0.01 ppm. Enhanced chemical shift perturbations in the α3′ turn could indicate that the Ppant arm has a preferred orientation towards this region. Alternatively, the backbone amide sites of residues 2117–2121 are all close to the side chain of Phe^2120^, which projects out from α3′ towards the attachment serine. A subtle change in side-chain χ_1_ rotamer populations caused by intermittent contacts with the prosthetic group could alter the ring current contribution to the chemical shifts of nearby sites sufficiently to account for the observed shift differences.

Additional derivatives were prepared by *in vitro* incubation of *apo* mACP_9_ with Sfp and various acyl coenzyme A thioesters. In this way malonyl, acetoacetyl, β-hydroxybutyryl, 2-butenoyl and butyryl groups were loaded on to *holo* mACP_9_ to mimic the various chemistries that occur during the reaction cycle of the MLSA2 module for a C_4_ chain (Figure 1A; Supplementary Figure S16). Comparison of the [^1^H,^15^N]-HSQC spectra of loaded mACP_9_ species with that of the *holo* form reveals that short acyl chains have little or no effect on the structure of the domain. Polar malonyl, acetoacetyl and β-hydroxybutyryl modifications yield only minor chemical shift differences (Figures 4B–4D), but when nonpolar 2-butenoyl and butyryl groups are attached to the prosthetic group small but significant (>0.06 ppm) perturbations are observed for sites in the α3′ turn (Figures 4E and 4F).

**Figure 5:**
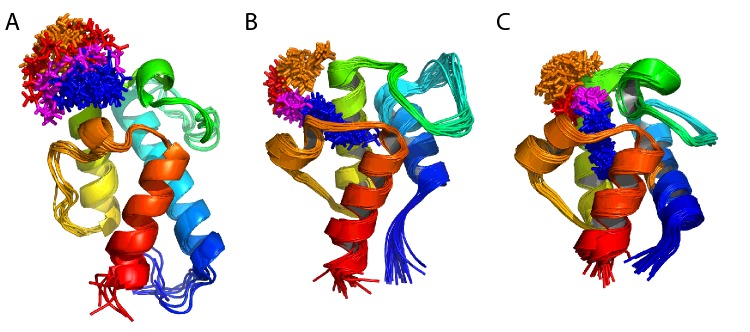
Solution structures of octanoyl-ACP species Ribbon representations, coloured from blue at the N-terminus to red at the C-terminus, for (**A**) octanoyl-mACP_9_ (5HV8); (**B**) octanoyl-actACP (2KGC); and (**C**) octanoyl-ACP from the *S. coelicolor* FAS (2KOS). Stick representations of pantoate, β-alanine, cysteamine and octanoyl sections are coloured orange, red, magenta and blue, respectively.

By contrast, longer, completely reduced hexanonyl and octanoyl chains cause much larger (>0.10 ppm) shift changes at backbone sites through the length of helix α2 to the end of the α3′ turn (Figures 4G and 4H). A ^12^C/^14^N-filtered ^13^C-separated NOESY-HSQC experiment on a sample in which the ACP domain was uniformly ^13^C/^15^N-labelled but the Ppant and octanoyl groups remained unlabelled revealed 26 NOE connections between the protein and the acyl chain. Together with 1949 protein–protein distance restraints, these were used to determine the solution structure of octanoyl-mACP_9_. The final ensemble has a backbone RMSD of 0.8 Å for residues 2054–2140 and the lowest energy structure is very close to that obtained for the *apo* state of mACP_9_ (backbone RMSD 1.1 Å). As Figure 5A shows, the Ppant moiety remains somewhat disordered (with a heavy atom RMSD of 3.4 Å), whereas the octanoyl group (heavy atom RMSD 1.3 Å) becomes more ordered towards the tip, which inserts into a small nonpolar pocket between helix α2 and the α2′ and α3′ turns.

Experiments following the change in mean residue ellipticity at 222 nm as a function of temperature demonstrated that *apo*, *holo*, hexanoyl- and octanoyl-mACP_9_ species all undergo two-state unfolding transitions, with melting temperatures of 326 K, 332 K, 331 K and 330 K respectively (Supplementary Figure S17). These results confirm earlier work on the type II FAS ACP from *P. falciparum*, which showed that priming with Ppant enhanced the thermal stability of the domain [41]. For mACP_9_, it is interesting that further modification with hexanoyl and octanoyl groups had minimal effects, rather than causing the melting temperature to increase. This suggests that the bound state captured in the solution structure of octanoyl-mACP_9_ is formed only transiently, with a lifetime long enough to allow for magnetization transfer effects when the substrate chain is close to the protein surface, but leaving the undocked state sufficiently populated that the free energy difference between the folded and denatured states of the domain is hardly affected.

In summary, the attachment of C_3_ and C_4_ chains has only a modest effect on the behaviour of the Ppant group, especially if the acyl groups are polar, whereas longer nonpolar C_6_ and C_8_ chains exceed a threshold for more significant, but still transient, interactions with a hydrophobic patch on the surface of the ACP domain.

### ^15^N nuclear spin relaxation studies

**Figure 6:**
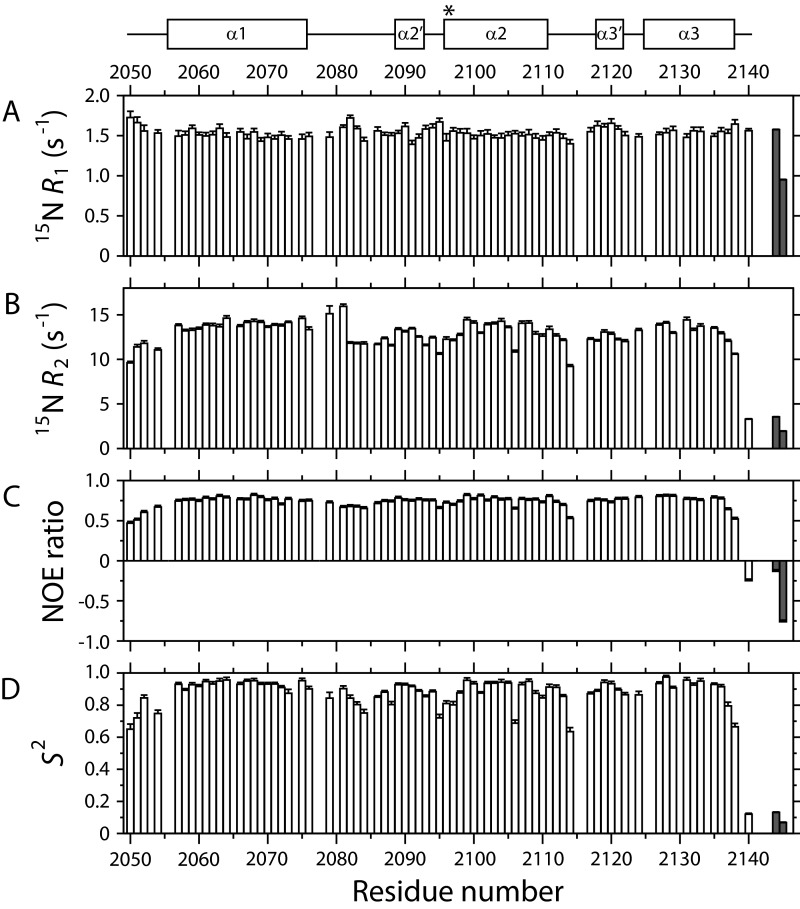
^15^N relaxation parameters for *holo* mACP_9_ Underneath a schematic defining the boundaries of α-helices in the structure of *apo* mACP_9_, NMR parameters for backbone and prosthetic group amide sites in the *holo* state are plotted as a function of residue number for: (**A**) the ^15^N longitudinal relaxation rate, *R*_1_; (**B**) the ^15^N transverse relaxation rate, *R*_2_; (**C**) the {^1^H}-^15^N NOE ratio (*I*′/*I*_0_, where *I*′ is the intensity when the ^1^H spectrum has been saturated and *I*_0_ is the intensity in the reference spectrum); and (**D**) the Lipari–Szabo the order parameter, *S*^2^. Bars shaded in grey correspond to values for the N^8^ (left) and N^4^ (right) sites of the prosthetic group.

To investigate the solution state dynamics of mACP_9_, nuclear spin relaxation properties were measured for ^15^N-labelled amide sites in both the *apo* and *holo* forms and analysed using the Lipari–Szabo model-free approach (Figure 6; Supplementary Figure S18). The data fitted best to an axially symmetric diffusion tensor model with a *D*_par_/*D*_per_ ratio of 1.30 and overall rotational correlation times of 10.3 ns and 10.4 ns for the *apo* and *holo* states, respectively, consistent with a monomeric, globular 90 amino acid domain at 283 K. In both the *apo* and *holo* states, backbone sites were found to be predominantly rigid (mean order parameter *S*^2^ of 0.87±0.10 for both), with the same five residues possessing more dynamic *S*^2^ values (<0.70): Met^2050^ at the N-terminus; Leu^2106^ in α2; Leu^2114^ in the loop between α2 and α3′ and Gln^2138^ and His^2140^ at the C-terminus. These similar relaxation properties indicate that no functionally relevant changes in backbone dynamics are induced on conversion of *apo* mACP_9_ to the *holo* form.

*In vivo* phosphopantetheinylation allowed relaxation parameters to be measured for the two ^15^N-labelled amide sites in the Ppant arm. Both nuclei displayed properties indicating elevated levels of local motion relative to the rest of the protein (see Table 2). For example, the heteronuclear NOE ratios for N^4^ (−0.76) and N^8^ (−0.13) possessed the opposite sign to the average value for *holo* protein backbone sites (+0.72±0.13). ^15^N *R*_1_ and *R*_2_ values for the Ppant sites were used to estimate effective local rotational correlation times (*τ*_eff_) of 3.7 ns and 4.1 ns for N^4^ and N^8^, respectively [37]. The relaxation properties of the Ppant sites were also analysed using the Lipari–Szabo model-free approach together with data from the rest of the *holo* state. As no structural data was available for the prosthetic group, an isotropic rotational diffusion model was assumed, which fit the data best using an overall correlation time of 10.9 ns and *S*^2^ order parameter values of 0.07 and 0.13 for N^4^ and N^8^ respectively. These low *S*^2^ values demonstrate that the Ppant moiety reorients much more rapidly than the rest of the protein and that the arm becomes significantly more dynamic as it extends outward from its point of attachment at the side chain of Ser^2096^. This behaviour is consistent with freely swinging motion in the prosthetic group, a conclusion supported by the observation that H^N4^ and H^N8^ display only weak, intra-Ppant NOE connections in the ^15^N-separated NOESY spectrum of *holo* mACP_9_. Our *in vitro* strategy for preparing acyl-loaded mACP_9_ samples relied on reactions between the *apo* protein and unlabelled coenzyme A derivatives; as a consequence, we did not have the opportunity to measure the relaxation properties of Ppant amide sites in acyl-ACP species.

**Table 2:** Dynamics parameters derived from ^15^N nuclear spin relaxation experiments

State	Species	*τ*_eff_ (ns)*	NOE ratio
*apo*	Backbone average	10.3	0.68
*holo*	Backbone average	10.5	0.72
*holo*	N^4^	3.7	–0.76
*holo*	N^8^	4.1	–0.13
ATSL, major	Backbone average	10.5	0.72
ATSL, major	N^4^	6.3	0.00
ATSL, major	N^8^	6.6	0.24
ATSL, minor	Backbone average	10.6	0.79
ATSL, minor	N^4^	5.3	0.00
ATSL, minor	N^8^	5.4	0.20

*Determined using the equation *τ*_eff_=[(6*R*_2_/*R*_1_) − 7]^0.5^/(2*ω*_N_) [37].

### Paramagnetic relaxation enhancement studies

**Figure 7:**
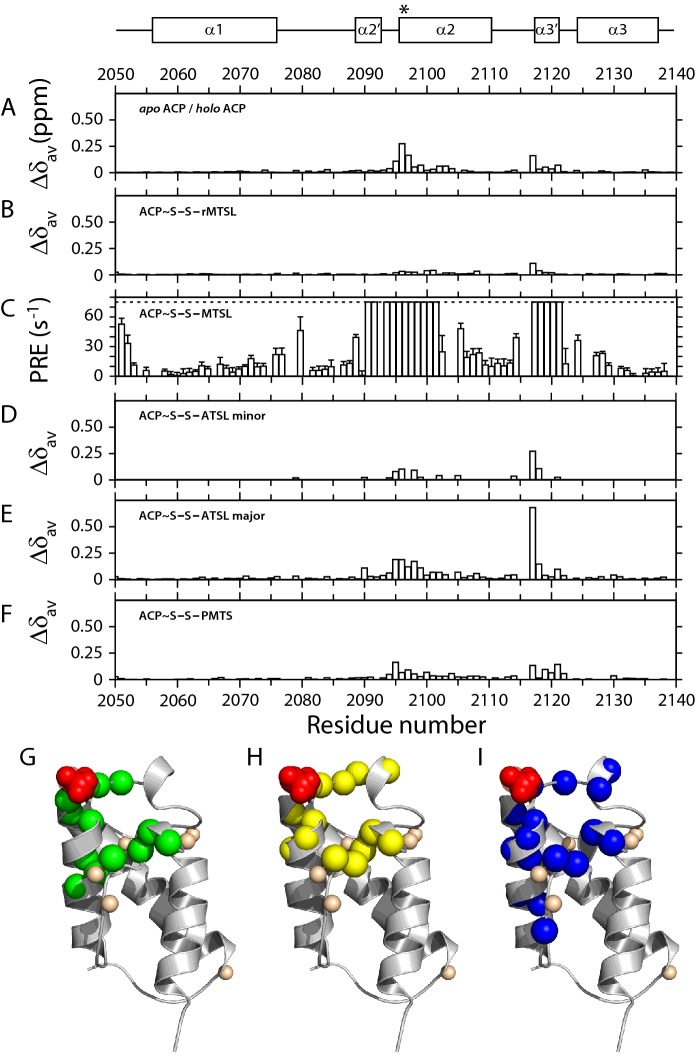
Average shift difference and PRE parameters for acyl-mACP_9_ species Underneath a schematic defining the boundaries of α-helices in the structure of *apo* mACP_9_, properties of disulfide-linked substrate mimics are plotted as a function of residue number. Average chemical shift differences, Δ*δ*_av_, are shown between *holo* mACP_9_ and (**A**) the *apo* form; (**B**) the reduced MTSL form; (**D**) the ATSL minor form (doubled peaks only); (**E**) the ATSL major form; and (**F**) the PMTS form. Panel (**C**) shows the PRE effects (defined as *R*_2,MTSL_ – *R*_2,ref_) for MTSL-mACP_9_. Cartoons of the *apo* mACP_9_ structure below highlight the Ser^2096^ modification site in red and amide sites that: (**G**) show significant shift changes between the *apo* and *holo* forms (green); (**H**) are bleached in the MTSL form (yellow); and (**I**) exhibit doubled resonances in ATSL-labelled mACP_9_ (blue). Small cream spheres indicate the nitrogen atoms of proline residues.

The chemical shift perturbations observed on conversion of mACP_9_ from the *apo* to the *holo* form could be interpreted as evidence that the Ppant group prefers to populate conformations that are oriented towards the α3′ turn, similar perhaps to those captured in the ensemble of structures for octanoyl-mACP_9_. By contrast, our ^15^N nuclear spin relaxation measurements suggest that the prosthetic group is highly flexible, implying that the arm samples multiple conformations in an isotropic fashion. To investigate this issue further, the Ppant arm of *holo* mACP_9_ was modified with the paramagnetic spin label reagent MTSL. Two reference samples were also prepared: one modified with ATSL, with the nitroxyl moiety replaced by an acetyl group (Supplementary Figure S16); and one in which the nitroxyl radical of MTSL was reduced to a diamagnetic hydroxy group via treatment with ascorbate. The ascorbate-reduced reference sample showed only minor chemical shift perturbations compared with non-MTSL-labelled *holo* mACP_9_ (Figure 7B), confirming that any interactions between the MTSL label and mACP_9_ must be similar to those observed for C_4_-loaded species.

Paramagnetic relaxation enhancements (PREs) were quantified as the difference between ^1^H^N^ transverse relaxation rates in the spin-labelled sample and in the reduced reference sample (*R*_2,MTSL_ − *R*_2,ref_) [40]. In the paramagnetic sample, several resonances exhibited greatly enhanced relaxation, to the extent that accurate *R*_2,MTSL_ measurements could not be made; to include these sites in Figure 7C, the value of *R*_2,MTSL_ − *R*_2,ref_ was set to an arbitrary maximum of 75 Hz. The relaxation enhancement profile is similar to that observed for chemical shift perturbations between the *apo* and *holo* forms (Figure 7A), with residues surrounding the attachment serine and around the α3′ turn affected most. As Figure 7H suggests, all amide signals from sites within 12.5 Å of the O^
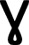
^ atom of Ser^2096^ were bleached or strongly broadened (including those from N^4^ and N^8^ in the Ppant arm). Proline residues lack an amide proton, so they are not probed by this technique.

### Modification with disulfide-linked groups

Unexpectedly, labelling of mACP_9_ with ATSL yielded a [^1^H,^15^N]-HSQC spectrum in which several resonances were doubled (Supplementary Figure S19), rendering the sample unsuitable for use as a reference state for PRE experiments. ESI MS revealed that the sample contained a single species at the expected molecular mass (Supplementary Figure S11), suggesting that the two forms detected by NMR correspond to distinct conformational states rather than alternative modification products. None of the doubled peaks coincide with resonances from the *apo* or *holo* forms of mACP_9_, indicating that modification with ATSL creates two significantly different conformations. All resolvable doublets comprise a minor peak with chemical shifts close to the corresponding *holo* signal (Figure 7D) and a major peak showing a larger shift perturbation (Figure 7E), with an intensity ratio of approximately 1:1.4. In *zz*-HSQC experiments, no chemical exchange cross-peaks between resolved doublets were detected (results not shown), implying that if the two states are capable of interconverting, this must occur on a timescale slower than 0.5 s. As Figure 7I shows, sites that give rise to doublets include those displaying the strongest PRE effects (Figure 7H), the largest chemical shift differences between the *apo* and *holo* forms (Figure 7G), and the most significant perturbations in 2-butenoyl- and butyryl-loaded samples, but affect fewer sites than those changed in an octanoyl-loaded sample. Amide signals from the prosthetic group were also doubled and displayed relaxation properties (*τ*_eff_ values of 6.5 ns and 5.4 ns for the major and minor forms, respectively; Table 2) intermediate between those of the protein backbone (∼10.5 ns) and the more dynamic arm of the *holo* species (∼3.9 ns). Consistent with these results, the H^N4^ and H^N8^ sites in both forms exhibited more intense ^1^H-^1^H NOEs than the *holo* state, although once again only intra-Ppant connections were observed.

Aiming to mimic the length of a butyryl substrate chain, we modified a sample of the *holo* domain using PMTS. The [^1^H,^15^N]-HSQC spectrum of PMTS-modified mACP_9_ showed a single set of resonances with chemical shift changes (Figure 7F) more extensive than those observed for butyryl-mACP_9_ (Figure 4F), but smaller than for either of the ATSL-modified forms (Figures 7D and 7E). Together with an absence of peak doubling for the reduced MTSL sample, these observations suggest that multiple ATSL-mACP_9_ states are a property of the altered nitroxide moiety rather than a consequence of the linkage mode, such as conformational isomerism of the disulfide bond [42]. The most likely explanation is that the ATSL-modified prosthetic group populates two distinct conformers, both of which dock against the surface of the ACP domain, differing perhaps in the orientation of the acetoxy moiety (Supplementary Figure S16). Within the Ppant group, motions are restricted compared with the freedom of movement experienced in the *holo* state, but a significant degree of flexibility is retained. Although ATSL is not a natural substrate for mACP_9_, these results provide a further demonstration that longer substrates can interact with the surface of a type I PKS ACP domain. Evidence that the prosthetic group possesses moderately restricted dynamics in both ATSL-loaded conformers is consistent with the picture conveyed by the solution structure of octanoyl-mACP_9_, in which the Ppant arm shows a degree of conformational heterogeneity that decreases as the acyl group approaches the protein surface.

## DISCUSSION

Although a wide variety of carrier protein domains have been studied previously [9], this report on MLSA2 from the mycolactone system is the first to compare the solution structures of both the *apo* and acyl-loaded forms of an ACP domain excised from a canonical type I PKS module. The only other published atomic resolution structure for an ACP from a type I modular PKS is for the *apo* domain from module 2 of the DEBS system (*ery*ACP_2_), which lacks DH and ER domains [43]. *Ery*ACP_2_ shares 48% sequence identity with mACP_9_ and its overall fold is highly similar, showing a backbone RMSD of 1.3 Å over residues equivalent to Leu^2054^-His^2140^ (Supplementary Table S3); the three main helices possess almost identical orientations, whereas the connecting loops and helical turns display a greater degree of variation. In contrast with the clustering of charged side chains towards one face observed for mACP_9_, the distribution of positive and negative charges is more even across the surface of *ery*ACP_2_ (Supplementary Figure S14). The mACP_9_ structure also resembles those of ACP domains from the CalE8 iterative type I PKS (backbone RMSD 1.1 Å), the curacin *trans*-AT hybrid PKS/NRPS system (1.5 Å), *S. coelicolor* type II FAS (1.8 Å), the actinorhodin type II PKS (2.0 Å) and rat type I FAS (2.3 Å) (Supplementary Table S3).

Some type II carrier proteins exhibit multiple slowly exchanging structural forms in solution [44–46] although such heterogeneity is apparently not ubiquitous [18,47]. The single sets of resonances observed in our [^1^H,^15^N]-HSQC experiments demonstrate that the *apo*, *holo* and acyl-loaded forms of mACP_9_ each appear to adopt a single conformational state on the timescale of milliseconds to seconds monitored by slow regime chemical exchange. The ^15^N relaxation properties of the *apo* and *holo* forms of mACP_9_ (Figure 6; Supplementary Figure S18) support this conclusion, showing that backbone amide sites are rigid on the sub-nanosecond and the micro- to milli-second timescales, except for close to the N- and C-termini and in the long linker region between helices α1 and α2. It therefore seems unlikely that chemical modification of mACP_9_ species would manipulate the populations of significantly different pre-existing protein conformations in order to optimize their surface structures for interactions with particular partner enzyme domains [44,48]. Similarly modest degrees of backbone flexibility have been detected in inter-helical regions for the CalE8 [49] and frenolicin type II PKS ACP domains [50].

The Ppant arms of *holo* ACP species adopt a spectrum of dynamic states, ranging from tightly bound to the carrier protein surface to freely swinging. For example, the prosthetic group of the *holo* form of an atypical *Geobacter metallireducens* ACP (*Gm*ACP_3_) is rigid, displaying *S*^2^ order parameter values similar to those for structured backbone sites (∼0.8), together with 23 Ppant-protein NOE connections [51]. The *Plasmodium falciparum* type II FAS ACP (*Pf*ACP) represents the centre ground, exhibiting two conformers in slow exchange due to different docked orientations of the Ppant moiety; in both states the prosthetic group is semi-rigid, with *S*^2^ values for N^4^ and N^8^ in the 0.1–0.5 range, but also 6 NOEs between the arm and the protein surface [46]. The prosthetic group of frenolicin *holo* ACP is at the high flexibility end of the scale, exhibiting chemical shifts identical with those for free coenzyme A, low *S*^2^ values (∼0.1) and no detectable Ppant-protein NOEs [50]. For *holo* mACP_9_, Ppant group behaviour is close to this freely swinging extreme, showing no evidence for NOEs to the protein surface and N^4^ and N^8^ order parameter values of 0.07 and 0.13, respectively (Table 2), consistent with the arm becoming progressively more dynamic as it protrudes into the solvent.

Interestingly, backbone ^1^H^N^/^15^N chemical shift perturbations appear to be less sensitive probes of protein/prosthetic group interactions than Ppant ^15^N relaxation parameter measurements or the detection of direct Ppant-protein NOE connections. For mACP_9_, which possesses a highly dynamic arm, the largest detected Δδ_av_ value between the *apo* and *holo* forms is ∼0.3 ppm (Figure 4A), whereas the largest corresponding shift change for the firmly bound *Gm*ACP_3_ species is only ∼0.4 ppm [51]. Furthermore, we found that the magnitude of shift changes between *apo* and *holo* mACP_9_ did not depend in a straightforward way on distance from the point of attachment of the prosthetic group.

We therefore modified the Ppant thiol group of *holo* mACP_9_ with a nitroxide spin label to determine whether PRE experiments could provide complementary information. As the reduced, diamagnetic form of MTSL-mACP_9_ shows no ^1^H^N^/^15^N shift perturbations from the *holo* species larger than 0.1 ppm (Figure 7B), we deduced that the motional properties of the prosthetic group are altered only to a minor extent by addition of the spin label. In [^1^H,^15^N]-HSQC spectra of the oxidized, paramagnetic form of MTSL-mACP_9_, all amide signals from sites close to Ser^2096^ either disappear or are strongly broadened (Figure 7C). It is not possible to interpret completely bleached signals in fine-grained detail, but we take the clustering of residues that experience substantial intensity reductions (Figure 7H) as an indication that each site is transiently approached by the paramagnetic centre to within 10 Å [40]. This result is consistent with the Ppant arm sampling a wide range of conformations in an isotropic manner, as would be expected for freely swinging motion. Similar conclusions were drawn in recent studies of an MTSL-modified *holo* peptidyl carrier protein from a teicoplanin-producing NRPS system [47].

In common with the structure of rat FAS ACP [14], *apo* mACP_9_ lacks an obvious central cavity, meaning that considerable rearrangement would be required to bury an acyl group in its hydrophobic core, in the style of type II carrier proteins [11]. When the *holo* form of rat FAS ACP was compared with species loaded with hexanoyl and palmitoyl chains, no ^1^H^N^/^15^N shift changes >0.05 ppm and no acyl-protein NOEs could be detected, leading to the conclusion that this type I domain does not sequester long hydrophobic chains from the solvent [14]. Likewise, no Δδ_av_ values >0.13 ppm were found between the *holo* and propionyl-, malonyl-, butyryl-, crotonyl- or hexanoyl-loaded species of *ery*ACP_2_ [16]. Derivatization of mACP_9_ with C_3_ and C_4_ acyl chains has a similar outcome, with no Δ*δ*_av_ values >0.15 ppm (Figure 4), suggesting that these substrate mimics remain predominantly exposed to the solvent. In contrast, weighted shift changes >0.5 ppm are apparent for both hexanoyl- and octanoyl-mACP_9_ species, alongside 26 NOE connections between the octanoyl chain and the protein surface.

The ensemble of solution structures determined for octanoyl-mACP_9_ shows the Ppant arm curling over to allow the acyl chain to nestle into a nonpolar pocket formed between the α2′ and α3′ turns and helix α2, whereas the prosthetic group itself remains solvent exposed (Figure 5A). This behaviour is similar to that observed for the ACP domain from the actinorhodin type II PKS (actACP), with no significant interactions detected between the protein and acetyl, malonyl, 3-oxobutyl or 3,5-dioxohexyl chains, but with large shift changes apparent for nonpolar butyryl, hexanoyl and octanoyl chains, yielding solution structures that showed Ppant and the acyl moieties rigidly docked into a groove between the α3′ turn and helices α2 and α3 (Figure 5B) [45]. For octanoyl-mACP_9_, the interaction of the acyl chain with the protein surface is clearly more superficial than that for octanoyl-actACP, which in turn does not penetrate as deeply into the hydrophobic core as seen in studies of octanoyl-ACP from the *S. coelicolor* type II FAS (Figure 5C) [12]. Furthermore, in the ensemble of structures for octanoyl-mACP_9_, the conformation of the Ppant moiety is relatively uncertain (RMSD 3.4 Å), implying that it retains a degree of flexibility analogous to that measured here for the major and minor states of ATSL-mACP_9_ (Table 2). This behaviour is different to the more precise definition of the prosthetic group in the octanoyl-actACP ensemble (RMSD 1.7 Å), which is restrained by 50 intra-Ppant, 12 Ppant-protein and 30 acyl-protein NOEs [45].

The NOE connection between a pair of nuclear spins corresponds to an *r*^−6^-weighted time and ensemble average over all the inter-nuclear distances sampled during the mixing time of a NOESY experiment [52]. This strong distance dependence rarely complicates the interpretation of NOE-based structures for folded protein domains, but it implies that structures of more dynamic systems may be biased towards conformations in which the two spins are close in space, even if these are populated sparsely and for relatively brief time periods. In this light, the ensemble of solution structures for octanoyl-mACP_9_ should be regarded as a collection of snapshots of bound conformations that provide no information about potential unbound states in which the acyl chain is fully exposed to solvent.

If a conformation in which a Ppant-tethered octanoyl group is bound to the surface of an ACP domain is highly populated, acyl-loading might be expected to stabilize the structure of the protein compared with the *holo* state, making it more resistant to thermal denaturation. To our surprise, when we used circular dichroism spectroscopy to monitor the unfolding of *apo*, *holo*, hexanoyl- and octanoyl-mACP_9_ species, acyl group attachment was found to have minimal effects on the melting temperature. By contrast, modification with Ppant increased the melting temperature of *apo* mACP_9_ by 6 K (Supplementary Figure S17). Phosphopantetheinylation of the *apo* state of *pf*ACP has been reported to cause a similar degree of stabilization, attributed to the formation of hydrophobic contacts between the prosthetic group and the protein surface [41]. In our hands, the Ppant group of *holo* mACP_9_ appears to swing freely around its attachment point, which suggests that other factors must be responsible for increasing its resistance to denaturation. Further studies will be needed to fully explain the effects of Ppant attachment on ACP domain thermostability, but the results of our NMR and circular dichroism experiments on octanoyl-mACP_9_ are consistent with its acyl chain making weak transitory interactions with the surface of the carrier protein.

Within the mycolactone PKS system, four other extender module ACP domains display >95% identity with mACP_9_: those from the third, seventh and eighth modules of MLSA1 and the seventh module of MLSB [25]. When mACP_9_ is included, this family of domains can be loaded with either malonyl or methylmalonyl extender units, whereas its natural substrate chains range in length from C_8_ to C_20_, can either possess or lack an α-branching methyl group, and can sample three of the four possible β-position oxidation states: an alcohol, an alkene or a fully reduced methylene group (Supplementary Figure S20). Compared with such diversity, this work has been able to characterize a limited panel of commercially available substrate-mimics (Supplementary Figure S16). Nevertheless, we have shown that when attached to a type I PKS ACP fragment short polar acyl chains remain exposed to the solvent, whereas longer, more saturated chains can interact (albeit transiently) with patches on the surface of the domain without significantly changing the structure of the protein.

In the context of an intact PKS module, our observations argue against channelling mechanisms that rely on substantial substrate-mediated structural modifications to optimize the shape of the ACP in such a way that protein–protein interactions with accessible partner domains are promoted, whereas others are prohibited. Alternatively, if the next destination of the ACP is to be decided by random diffusion between a pre-selected subset of partner domains [10] followed by recognition of substrate chemistry alone, this would presumably be favoured by arranging for solvent exposed acyl cargoes to be presented by highly dynamic Ppant arms. Despite an apparent concordance between this mechanism and our results for short polar substrate-mimics, the sub-states identified in cryo-EM experiments on Pik module 5 suggest that both protein–protein interfaces and correct substrate chemistry are required for the ACP to find an appropriate active site [20,21]. Unfortunately, the >7 Å resolution of these cryo-EM studies was not sufficient to localize any Ppant or acyl moieties, so the relative importance of interactions with the ACP surface, the prosthetic group or the substrate itself has not yet been established for any of the detected sub-states.

Although not obtained using a natural substrate, our findings for octanoyl-mACP_9_ pose the intriguing possibility that a partner domain may be able to recognize an acyl group whereas it is bound to the surface of its carrier protein, allowing simultaneous interactions with both the substrate and the ACP. Subtle variations in the substrate chemistry, ACP-docked conformation and features of the adjacent ACP surface could then favour encounters with the correct partner in order to facilitate progress through the reaction cycle. From this perspective, the apparent flexibility of the Ppant and acyl portions of octanoyl-mACP_9_ need not be seen as a disadvantage, as a conformational selection mechanism triggered by proximity to the cognate surface of a partner domain could permit the substrate/Ppant/ACP interface to adapt so as to promote productive interactions with the appropriate active site. Similar mechanisms have recently been suggested for substrate-loaded NRPS aryl and peptidyl carrier protein domains from the Type I yersiniabactin [18] and the Type II pyoluteorin [19] systems.

Various novel methods have been used to investigate Ppant behaviour, including the attachment of solvatochromic groups for fluorescence spectroscopy [53], a cyanyl group for vibrational spectroscopy [54] and fluorophenyl [55] or trifluoromethyl [16] substituted acyl groups for ^19^F NMR spectroscopy. These approaches were primarily designed to sense whether the probe was buried in a nonpolar environment or exposed to solvent, and do not return information about Ppant dynamics or details about any detected site of interaction. Moreover, all three methods require covalent modification of the Ppant arm, making them incompatible with studies of natural acyl-ACP species, as well as potentially perturbing the dynamics and interaction motifs of the prosthetic group. This work has deployed NOE-based structure determination, ^1^H^N^/^15^N chemical shift perturbation, nuclear spin relaxation and PRE as a suite of tools that in combination can report directly on the conformational and dynamic properties of ACP-attached and substrate-loaded Ppant groups. Having demonstrated that the Ppant arm in some circumstances swings freely, but in others is ‘sticky’ enough to interact with the surface of the carrier protein, the logical next step is to characterize the role of prosthetic group dynamics in binary interactions between ACP and partner domain fragments and within an intact PKS module.

## Abbreviations

ACP: acyl carrier protein
AT: acyltransferase
ATSL: (1-acetoxy-2,2,5,5-tetramethyl-∆3-pyrroline-3-methyl) methanethiosulfonate
cryo-EM: cryo-electron microscopy
DH: dehydratase
ER: enoyl reductase
ESI MS: electrospray injection mass spectrometry
FAS: fatty acid synthase
HSQC: heteronuclear single quantum coherence
KR: ketoreductase
KS: ketosynthase
MTSL: (1-oxyl-2,2,5,5-tetramethyl-∆3-pyrroline-3-methyl) methanethiosulfonate
NRPS: non-ribosomal peptide synthetase
PKS: polyketide synthase
PMTS: *n*-propyl methanethiosufonate
Ppant: 4′-phosphopantetheine
PRE: paramagnetic relaxation enhancement
TE: thioesterase

## References

[1] Staunton J., Weissman K.J. (2001). Polyketide biosynthesis: a millennium review. Nat. Prod. Rep..

[2] Keatinge-Clay A.T. (2012). The structures of type I polyketide synthases. Nat. Prod. Rep..

[3] Weissman K.J., Leadlay P.F. (2005). Combinatorial biosynthesis of reduced polyketides. Nat. Rev. Microbiol..

[4] Kim E., Moore B.S., Yoon Y.J. (2015). Reinvigorating natural product combinatorial biosynthesis with synthetic biology. Nat. Chem. Biol..

[5] Floss H.G. (2006). Combinatorial biosynthesis: potential and problems. J. Biotechnol..

[6] Poust S., Hagen A., Katz L., Keasling J.D. (2014). Narrowing the gap between the promise and reality of polyketide synthases as a synthetic biology platform. Curr. Opin. Biotechnol..

[7] Khosla C., Herschlag D., Cane D.E., Walsh C.T. (2014). Assembly line polyketide synthases: mechanistic insights and unsolved problems. Biochemistry.

[8] Weissman K.J., Müller R. (2008). Protein–protein interactions in multienzyme megasynthetases. ChemBioChem.

[9] Crosby J., Crump M.P. (2012). The structural role of the carrier protein–active controller or passive carrier. Nat. Prod. Rep..

[10] Weissman K.J. (2015). The structural biology of biosynthetic megaenzymes. Nat. Chem. Biol..

[11] Cronan J.E. (2014). The chain-flipping mechanism of ACP (acyl carrier protein)-dependent enzymes appears universal. Biochem. J..

[12] Płoskoń E., Arthur C.J., Kanari A.L.P., Wattana-amorn P., Williams C., Crosby J., Simpson T.J., Willis C.L., Crump M.P. (2010). Recognition of intermediate functionality by acyl carrier protein over a complete cycle of fatty acid biosynthesis. Chem. Biol..

[13] Tran L., Broadhurst R.W., Tosin M., Cavalli A., Weissman K.J. (2010). Insights into protein–protein and enzyme–substrate interactions in modular polyketide synthases. Chem. Biol..

[14] Płoskoń E., Arthur C.J., Evans C., Williams C., Crosby J., Simpson T.J., Crump M.P. (2008). A mammalian type I fatty acid synthase acyl carrier protein domain does not sequester acyl chains. J. Chem. Biol..

[15] Wattana-amorn P., Williams C., Płoskoń E., Cox R.J., Arthur C.J., Simpson T.J., Crosby J., Crump M.P. (2010). Solution structure of an acyl carrier protein domain from a fungal type I polyketide synthase. Biochemistry.

[16] Charkoudian L.K., Liu C.W., Capone S., Kapur S., Cane D.E., Togni A., Seebach D., Khosla C. (2011). Probing the interactions of an acyl carrier protein domain from the 6-deoxyerythronolide B synthase. Protein Sci..

[17] Nguyen C., Haushalter R.W., Lee D.J., Markwick P.R.L., Bruegger J., Caldera-Festin G., Finzel K., Jackson D.R., Ishikawa F., O'Dowd B. (2014). Trapping the dynamic acyl carrier protein in fatty acid biosynthesis. Nature.

[18] Goodrich A.C., Harden B.J., Frueh D.P. (2015). Solution structure of a nonribosomal peptide synthetase carrier protein loaded with its substrate reveals transient, well-defined contacts. J. Am. Chem. Soc..

[19] Jaremko M.J., Lee J.D., Opella S.J., Burkart M.D. (2015). Structure and substrate sequestration in the pyoluteorin peptidyl carrier protein PltL. J. Am. Chem. Soc..

[20] Dutta S., Whicher J.R., Hansen D.A., Hale W.A., Chemler J.A., Congdon G.R., Narayan A.R., Håkansson K., Sherman D.H., Smith J.L., Skiniotis G. (2014). Structure of a modular polyketide synthase. Nature.

[21] Whicher J.R., Dutta S., Hansen D.A., Hale W.A., Chemler J.A., Dosey A.M., Narayan A.R., Håkansson K., Sherman D.H., Smith J.L., Skiniotis G. (2014). Structural rearrangements of a polyketide synthase module during its catalytic cycle. Nature.

[22] Weissman K.J. (2015). Uncovering the structures of modular polyketide synthases. Nat. Prod. Rep..

[23] George K.M., Pascopella L., Welty D.M., Small P.L. (2000). A *Mycobacterium ulcerans* toxin, mycolactone, causes apoptosis in guinea pig ulcers and tissue culture cells. Infect. Immun..

[24] Bali S., Weissman K.J. (2006). Ketoreduction in mycolactone biosynthesis: insight into substrate specificity and stereocontrol from studies of discrete ketoreductase domains *in vitro*. ChemBioChem..

[25] Stinear T.P., Mve-Obiang A., Small P.L., Frigui W., Pryor M.J., Brosch R., Jenkin G.A., Johnson P.D., Davies J.K., Lee R.E. (2004). Giant plasmid-encoded polyketide synthases produce the macrolide toxin of *Mycobacterium ulcerans*. Proc. Natl. Acad. Sci. U.S.A..

[26] Sambrook J., Russell D.W. (2001). Molecular Cloning: A Laboratory Manual.

[27] Martinez E., Bartolomé B., de la Cruz F. (1998). pACYC184-derived cloning vectors containing the multiple cloning site and lacX alpha reporter gene of pUC8/9 and pUC18/19 plasmids. Gene.

[28] Quadri L.E., Weinreb P.H., Lei M., Nakano M.M., Zuber P., Walsh C.T. (1998). Characterization of Sfp, a *Bacillus subtilis* phosphopantetheinyl transferase for peptidyl carrier protein domains in peptide synthetases. Biochemistry.

[29] Cavanagh J., Fairbrother W.J., Palmer A.G., Skelton N.J. (2006). Protein NMR Spectroscopy: Principles and Practice.

[30] Vranken W.F., Boucher W., Stevens T.J., Fogh R.H., Pajon A., Llinas M., Ulrich E.L., Markley J.L., Ionides J., Laue E.D. (2005). The CCPN data model for NMR spectroscopy: development of a software pipeline. Proteins.

[31] Pellecchia M., Sebbel P., Hermanns U., Wuthrich K., Glockshuber R. (1999). Pilus chaperone FimC-adhesin FimH interactions mapped by TROSY-NMR. Nat. Struct. Biol..

[32] Bardiaux B., Bernard A., Rieping W., Habeck M., Malliavin T.E., Nilges M. (2009). Influence of different assignment conditions on the determination of symmetric homodimeric structures with ARIA. Proteins.

[33] Cheung M.S., Maguire M.L., Stevens T.J., Broadhurst R.W. (2010). DANGLE: a Bayesian inferential method for predicting protein backbone dihedral angles and secondary structure. J. Magn. Reson..

[34] Sousa da Silva A.W., Vranken W.F. (2012). ACPYPE–antichamber python parser interface. BMC Res. Notes.

[35] Wang J., Wolf R.M., Caldwell J.W., Kollman P.A., Case D.A. (2004). Development and testing of a general AMBER force field. J. Comput. Chem..

[36] Wang J., Wang W., Kollman P.A., Case D.A. (2006). Automatic atom type and bond type perception in molecular mechanical calculations. J. Mol. Graph. Model..

[37] Kay L.E., Torchia D., Bax A. (1989). Backbone dynamics of proteins as studied by ^15^N inverse detected heteronuclear NMR spectroscopy: application to staphylococcal nuclease. Biochemistry.

[38] Palmer A., Rance M., Wright P. (1991). Intramolecular motions of a zinc finger DNA-binding domain from Xfin characterized by proton-detected natural abundance carbon-13 heteronuclear NMR. J. Am. Chem. Soc..

[39] Mandel A.M., Akke M., Palmer A.G. (1995). Backbone dynamics of *Escherichia coli* ribonuclease HI: correlations with structure and function in an active enzyme. J. Mol. Biol..

[40] Clore G.M., Iwahara J. (2009). Theory, practice and applications of paramagnetic relaxation enhancement for the characterization of transient low-population states of biological macromolecules and their complexes. Chem. Rev..

[41] Modak R., Sinha S., Surolia N. (2007). Isothermal unfolding studies on the apo and holo forms of *Plasmodium falciparum* acyl carrier protein: role of the 4′-phosphopantetheine group in the stability of the *holo* form of the *Plasmodium falciparum* acyl carrier protein. FEBS J..

[42] Grey M.J., Wang C., Palmer A.G. (2003). Disulphide bond isomerisation in basic pancreatic trypsin inhibitor: multisite chemical exchange quantified by CPMG relaxation dispersion and chemical shift modelling. J. Am. Chem. Soc..

[43] Alekseyev V.Y., Liu C.W., Cane D.E., Puglisi J.D., Khosla C. (2007). Solution structure and proposed domain domain recognition interface of an acyl carrier protein domain from a modular polyketide synthase. Protein Sci..

[44] Koglin A., Mofid M.R., Löhr F., Schäfer B., Rogov V.V., Blum M.M., Mittag T., Marahiel M.A., Bernhard F., Dötsch V. (2006). Conformational switches modulate protein interactions in peptide antibiotic synthetases. Science.

[45] Evans S.E., Williams C., Arthur C.J., Płoskoń E., Wattana-amorn P., Cox R.J., Crosby J., Willis C.J., Simpson T.J., Crump M.P. (2009). Probing the interactions of early polyketide intermediates with the actinorhodin ACP from *S. coelicolor* A3(2). J. Mol. Biol..

[46] Sharma A.K., Sharma S.K., Surolia A., Surolia N., Sarma S.P. (2006). Solution structures of conformationally equilibrium forms of holo-acyl carrier protein (PfACP) from *Plasmodium falciparum* provides insight into the mechanism of activation of ACPs. Biochemistry.

[47] Haslinger K., Redfield C., Cryle M. (2015). Structure of the terminal PCP domain of the non-ribosomal peptide synthetase in teicoplanin biosynthesis. Proteins.

[48] Tufar P., Rahighi S., Kraas F.I., Kirchner D.K., Löhr F., Henrich E., Köpke J., Dikic I., Güntert P., Marahiel M.A., Dötsch V. (2014). Crystal structure of a PCP/Sfp complex reveals the structural basis for carrier protein posttranslational modification. Chem. Biol..

[49] Lim J., Kong R., Murugan E., Ho C.L., Liang Z.X., Yang D. (2011). Solution structures of the acyl carrier protein domain from the highly reducing type I iterative polyketide synthase CalE8. PloS One.

[50] Li Q., Khosla C., Puglisi J.D., Liu C.W. (2003). Solution structure and backbone dynamics of the *holo* form of the frenolicin acyl carrier protein. Biochemistry.

[51] Ramelot T.A., Smola M.J., Lee H.W., Ciccosanti C., Hamilton K., Acton T.B., Xiao R., Everett J.K., Prestegard J.H., Montelione G.T., Kennedy M.A. (2011). Solution structure of 4′-phosphopantetheine-GmACP3 from *Geobacter metallireducens*: a specialized acyl carrier protein with atypical structural features and a putative role in lipopolysaccharide biosynthesis. Biochemistry.

[52] Zagrovic B., van Gunsteren W.F. (2006). Comparing atomistic simulation data with NMR experiment: how much can NOEs actually tell us?. Proteins.

[53] Beld J., Cang H., Burkart M.D. (2014). Visualizing the chain-flipping mechanism in fatty-acid biosynthesis. Angew. Chem. Int. Ed..

[54] Johnson M.N.R., Londergan C.H., Charkoudian L.K. (2014). Probing the phosphopantetheine arm conformations of acyl carrier proteins using vibrational spectroscopy. J. Am. Chem. Soc..

[55] Lloyd R.P. (2014). Structural Studies of Acylated Forms of an Acyl Carrier Protein from *Saccharopolyspora erythraea*. Ph.D. thesis.

